# Unveiling Rare Pathogens and Antibiotic Resistance in Tanzanian Cholera Outbreak Waters

**DOI:** 10.3390/microorganisms11102490

**Published:** 2023-10-04

**Authors:** Vito Baraka, Tilde Andersson, Geofrey Makenga, Filbert Francis, Daniel T. R. Minja, Sören Overballe-Petersen, Man-Hung Eric Tang, Kurt Fuursted, Rolf Lood

**Affiliations:** 1 Tanga Centre, National Institute for Medical Research, Tanga P.O. Box 5004, Tanzania; vitobaraka@gmail.com (V.B.); geofmacky@gmail.com (G.M.); ffrancis8@gmail.com (F.F.); minjartd@gmail.com (D.T.R.M.); 2 Department of Clinical Sciences, Lund University, 221 84 Lund, Sweden; tilde.andersson@uzh.ch; 3 Bacterial Reference Center, Statens Serum Institut, 2300 Copenhagen, Denmark; soren@nanopore.dk (S.O.-P.); kfu@ssi.dk (K.F.); 4 Department of Bacteria, Statens Serum Institut, Parasites and Fungi, 2300 Copenhagen, Denmark; mhet@ssi.dk

**Keywords:** bacteriophage, antibiotic resistance, transduction, One Health, ESBL, microbiota, water, cholera, zoonotic pathogen

## Abstract

The emergence of antibiotic resistance is a global health concern. Therefore, understanding the mechanisms of its spread is crucial for implementing evidence-based strategies to tackle resistance in the context of the One Health approach. In developing countries where sanitation systems and access to clean and safe water are still major challenges, contamination may introduce bacteria and bacteriophages harboring antibiotic resistance genes (ARGs) into the environment. This contamination can increase the risk of exposure and community transmission of ARGs and infectious pathogens. However, there is a paucity of information on the mechanisms of bacteriophage-mediated spread of ARGs and patterns through the environment. Here, we deploy Droplet Digital PCR (ddPCR) and metagenomics approaches to analyze the abundance of ARGs and bacterial pathogens disseminated through clean and wastewater systems. We detected a relatively less-studied and rare human zoonotic pathogen, *Vibrio metschnikovii*, known to spread through fecal-–oral contamination, similarly to *V. cholerae*. Several antibiotic resistance genes were identified in both bacterial and bacteriophage fractions from water sources. Using metagenomics, we detected several resistance genes related to tetracyclines and beta-lactams in all the samples. Environmental samples from outlet wastewater had a high diversity of ARGs and contained high levels of *blaOXA-48*. Other identified resistance profiles included *tetA*, *tetM*, and *blaCTX-M9*. Specifically, we demonstrated that *blaCTX-M1* is enriched in the bacteriophage fraction from wastewater. In general, however, the bacterial community has a significantly higher abundance of resistance genes compared to the bacteriophage population. In conclusion, the study highlights the need to implement environmental monitoring of clean and wastewater to inform the risk of infectious disease outbreaks and the spread of antibiotic resistance in the context of One Health.

## 1. Introduction

Emergence and spread of antimicrobial resistance (AMR) pose a serious global public health threat and could impact economic growth. AMR infections contribute to approximately 700,000 deaths annually [1]. However, the burden of AMR is known to be heterogeneous and varies considerably between different regions. The World Bank projected that, without action, mortality related to AMR could surge up to 10 million annually by 2050 and cause approximately a 3.8 percent reduction in annual gross domestic product (GDP) [2]. The study demonstrated that sub-Saharan Africa has the highest burden of AMR, associated with 23.5 deaths per 100,000, attributable to multidrug-resistant pathogens [2].

Tanzania has established a comprehensive national action plan on antibiotic resistance (2017 to 2022) [3] and the current updated plan for 2023–2028 [4]. The action plan emphasizes the principle of addressing antimicrobial resistance under the concepts of the One Health approach (OH) to inform evidence-based strategies for AMR monitoring, prevention, and containment. The OH concepts recognize the multisectoral interconnectedness between human, animal, and environmental health [5,6]. In line with the plan, the country has established AMR surveillance through national sentinel sites to generate nationwide data and inform policies and practices. However, despite the existence of the action plan, the operationalization of OH strategies remains challenging due to several reasons, including limited intersectoral collaboration and coordination, financial constraints, and inadequate awareness of AMR and OH concepts.

Multidrug resistance (MDR) in bacterial infections has been shown to be a major public health concern in developing countries [2]. The emergence and spread of MDR pathogens are associated with the overuse of antibiotics in humans, poultry, fishing, and livestock production, poor-quality antibiotics, poor compliance with prescribed antibiotics, and access-related issues [5,7,8]. Studies in Tanzania suggest a high level of resistance to commonly used antibiotics, including ampicillin, cotrimoxazole, gentamicin, and chloramphenicol among *S. aureus*, *E. coli*, and *P. aeruginosa* [9,10,11,12]. Alarmingly, studies have shown a high proportion of resistant Gram-negative bacteria to third-generation (ceftriaxone/cefotaxime) and fourth-generation (cefepime) cephalosporins in clinical settings [9]. The high rate of antibiotic resistance is associated with the overprescription of antibiotics in clinical settings for treating MDR infections and could disseminate from clinical settings to the environment [10,12,13].

Potential environmental exposure to AMR and pathogenic agents includes water from wells, rivers, and lakes, recreational water, drinking water, crop irrigation using contaminated water, wastewater from hospitals and industries, and the consumption of contaminated food. In most resource-constrained settings where access to quality and clean water is limited, and sanitation systems are poorly developed, there may be an increased risk of exposure. The spread of AMR and ARGs in the environment is poorly understood in Tanzania, as in other low- and middle-income countries (LMICs), and the role of the environment in AMR transmission remains unclear. On the other hand, despite a tremendous increase in access to water supply, sanitation, and hygiene services, only 61% of households in Tanzania have access to basic water supply, 32% have access to basic sanitation, and 48% have access to basic hygiene [14]. Due to poor access to clean water, hygiene practices, and poor sanitation systems, microbial contamination of freshwater sources is a contributing factor. As a result, waterborne bacterial infections due to *Campylobacter* spp., *Shigella* spp., *Yersinia* spp., toxin-producing *E. coli* bacteria, *Salmonella* spp., and *Vibrio cholerae* are a common cause of diarrhea and gastroenteritis. Additionally, waterborne diseases due to parasitic and viral causes are common due to contamination of water sources. Cholera is endemic in Tanzania, with recent data showing 72 cases (3 deaths) over the last year. Previously, a large outbreak occurred in 1997, causing a total of 40,249 cases (2231 deaths) [15]. Communities with poor access to clean water rely on rivers, lakes, ponds, open wells, and irrigation canals as their main sources of clean water. However, common unhygienic practices such as open defecation in the environment, rivers, and surrounding bodies of water, bathing at water sources, poor sanitation, agricultural runoff, industrial, and domestic pollution lead to water contamination [14]. Another major environmental threat that triggers disease outbreaks is the concern for the impact of global climate change on public health. Climatic factors have been shown to correlate with the increased incidence of foodborne and waterborne diseases due to changes in weather parameters such as increasing ambient temperature and high or low rainfall [16]. Extreme weather events can lead to flooding and droughts, which can affect the concentration of pathogens through alterations in nutrient concentrations, salinity, and pH, impacting microbial survival [17]. During rainfall seasons, the risk of microbial contamination can increase due to runoff that may carry fecal materials contaminated by enteric pathogens, posing risks of waterborne diseases. These practices potentially contribute to frequent outbreaks of waterborne diseases for downstream communities using the water sources for drinking and domestic purposes. Additionally, there is increasing evidence of the association between high temperatures and increased antibiotic resistance in bacterial pathogens, including the common pathogens *E. coli*, *Klebsiella pneumoniae*, and *S. aureus* [18].

In addition, water source contamination may contribute to horizontal gene transfer (HGT) of mobile genetic elements (MGEs) and antibiotic resistance genes (ARGs) from clinical settings to environmental bacteria, promoting the accumulation and dissemination of ARGs in Gram-negative and Gram-positive bacteria [19,20]. It is well established that antibiotic resistance in bacteria may emerge from chromosomal genetic changes [7]. However, it has been shown that the acquisition of ARGs mediated by HGT mechanisms is a more common mechanism of resistance in bacteria [21]. Recently, transduction by bacteriophages has gained attention as an important mechanism in spreading ARGs and virulence genes within a microbial population [22,23]. ARG-carrying bacteriophages may accumulate in water organisms, potentially entering the food chain. Communities using the water for domestic and recreational use may be exposed to ARG-carrying bacteriophages, which, once ingested, may transfer ARGs to the gut microbiota, causing community-acquired resistant infections [24]. Viriome studies have shown that phages may contribute to antibiotic resistance because prophages were able to carry ARGs, and both lytic and lysogenic phages were demonstrated to transfer ARGs by transduction [20,22]. Studies using human fecal materials have demonstrated a high abundance of ARGs among phageomes. In other studies, human microbial tracking showed that they harbor at least one ARG, including *blaTEM*, *blaCTX-M-1*, *mecA*, *armA*, *qnrA*, and *qnrS* [25]. Interestingly, phage biomarkers identified as crAssphage were shown to correlate well with the persistence, fate, and possible selection of resistance genes due to fecal contamination, which is likely the main source of resistance genes in environmental ARG pollution. This potentially provides a quick, easy, and affordable way to monitor environmental pollution [26]. The evidence suggests that environments where sanitation and hygiene are poorly managed and characterized by fecal contamination in sewage, rivers, and effluent of wastewater treatment systems could play a role in the dissemination of microbial resistance as hotspots for bacterial genomes and phageomes harboring ARGs. Environmental surveillance of AMR to identify pathogens, potential mechanisms, pathways, and ARGs is critical to ensure effective AMR mitigation strategies using the One Health concept.

Water-based genomic epidemiology surveillance has demonstrated potential when used to investigate the emergence of antimicrobial resistance, surveillance, and early detection of disease outbreaks [27]. Harnessing technological advances in the field of next-generation sequencing (NGS) has significantly revolutionized the surveillance of disease pathogens and AMR in the environment and clinical settings [28]. Using metagenomics tools, it is possible to overcome the obstacles imposed by conventional culture-based methods to detect pathogens with outbreak potential and to improve the identification of environmental antibiotic resistance genes, consensus and non-coding sequences associated with ARGs and MGEs in the context of OH [29]. Hence, this study focuses on leveraging the use of genomics tools to characterize bacterial pathogens and bacteriophage ARG profiles from environmental samples collected from clean and wastewater sites in selected areas of the Tanga region in northeastern Tanzania.

## 2. Materials and Methods

### 2.1. Collection of Environmental and Clean Water Samples

**Figure 1:**
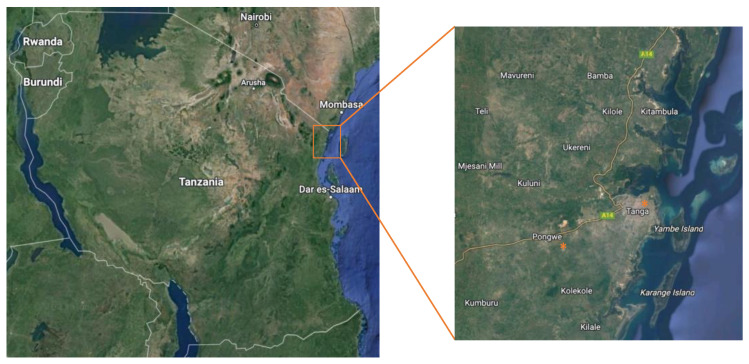
Schematic representation of sampling areas in Tanzania. Water sources from Tanga city and the nearby village Pongwe (orange dots) were collected, according to the defined geographical locations (Table 1).

All the materials were purposefully collected in June 2019 during a local cholera outbreak in the Pongwe district, outside of Tanga, and at the Tanga Regional Referral Hospital (TRRH) located in Tanga city, Tanzania (Figure 1). The cholera outbreak itself was coincidental but allowed for interesting samples. In Pongwe, samples were collected from the supposed sources (e.g., a contaminated well outside the village of Maranzara that by local health organizations had been assumed, but not verified, as the source of the outbreak (Pcc); the well itself has a tap-based system attached to it, from where the water was taken), neighboring water sources (tap and well; Pt#1, Pt#2, Pc#1, Pc#2), as well as from different clean water sources at the local hospital in Tanga (Th, Tmw), and wastewater (Tww#1, Tww#2). All samples were collected as single samples from each region, at one time point only (e.g., all samples were collected on the same day, and Pt#1 equals one sample, Pt#2 another sample, etc.). Due to the descriptive nature of the study, it was determined that it was more beneficial for the study to sample a larger representative volume, than three smaller individual samples, even though the power of the study may be negatively affected. Further, since most samples were tap-based, there should be very limited variation between samples collected at the same time. The distance between Tanga and Pongwe is approximately 15 km, while the distance between the individual samples within the Pongwe district was less than 1 km. Samples collected from wells were not connected to public water supplies. Wastewater samples from the hospital were collected from a manhole receiving direct sewage from each block or facility. Wastewater or clean water was collected from each hospital block or facility using sterile plastic bottles (Th, Tmw). Additionally, we collected samples from the main manhole discharging mixed wastewater from the entire hospital at two different sites (Tww#1, Tww#2). Samples were preserved on ice and transported to the central processing laboratory within 2 h. The collected samples were aliquoted into four 50 mL samples for phage DNA, bacterial DNA, antibiotic concentration, and backup specimens. All samples were collected during the weekdays, and GPS (Global Positioning System) coordinates and temperatures were recorded. Due to the low presence of microbes in some water samples, certain downstream analyses could not be performed on all samples.

### 2.2. Ethical Considerations

Ethical clearance was obtained from the Medical Research Coordinating Committee (MRCC) of the National Institute for Medical Research (NIMR MRCC): (NIMR MRCC; reference number NIMR/HQ/R8.a/Vol.IX/3079). In addition, the facility in-charge, village and ward leaders, as well as the district and regional medical authorities, were informed and approved the study in their settings.

### 2.3. Preparation of DNA for Bacteria and Bacteriophages

Water samples extracted from the locations given in Figure 1 (50 mL) were centrifuged to collect all cellular material, and bacterial DNA was extracted and purified using the PureLink Microbiome DNA purification kit (Invitrogen, Grand Island, NY, USA) following the manufacturer’s instructions. For bacteriophage samples, water samples were sterile-filtered and concentrated through 100 k MWCO filters. DNA was extracted and purified using a phage DNA isolation kit (Norgen Biotek, Thorold, ON, Canada), according to the manufacturer’s instructions. Afterward, DNA was quantified with a Nanodrop ND1000 and stored at −20 °C until further analyses.

### 2.4. 16S/18S Amplicon-Based Microbiome Analysis

Library preparation was performed using the Nextera XT DNA Library Preparation kit (Illumina Inc., San Diego, CA, USA), and Illumina sequencing was conducted on the HiSeq platform (Illumina) following the manufacturer’s instructions. DNA was amplified using a two-step PCR with custom 341F/806R primers targeting the V3–V4 16S regions, and three primer sets targeting the hyper-variable regions V3–V4 of the 18S rDNA gene. Amplicons were then sequenced on the Illumina MiSeq (Illumina) using the V2 Reagent Kit.

### 2.5. Oxford Nanopore Sequencing for Resistance Genes

Sequencing was conducted as previously described [30,31,32]. In brief, DNA was prepared for sequencing using the Oxford Nanopore Technologies’ Rapid PCR Barcoding Kit (SQK-RPB004) with the following modifications to the manufacturer’s instructions: double the volume of template DNA and ‘FRM’, as well as 25 PCR cycles instead of 14 cycles for improved sensitivity. DNA libraries were sequenced using a MinION Mk1B (Oxford Nanopore Technologies, Oxford, UK) connected to a MinIT with MinIT Release 19.12.5 (MinKNOW Core 3.6.5, Bream 4.3.16, Guppy 3.2.10).

### 2.6. Bioinformatics Analysis of Sequence Data

Bioinformatics was done using BION (https://tinyurl.com/3fn8hvaj, accessed on 20 March 2021), a newly developed analytical semi-commercial open-source package for 16S rRNA and other reference gene analysis, classifying mostly to species level.

### 2.7. Statistics of Sequence Data

The analysis of microbiome composition was performed in R version 4.0.3 (10 October 2020) using the packages phyloseq v.1.24.2 and vegan v.2.5-2 [30]. Figures were created using ggplot2 v.3.2.0 and plotly v.4.8.0. In the bar plot, taxa were merged at the genus level by aggregating counts within each genus. Alpha-diversity of samples, as well as the relative abundances of individual genera, were compared between groups using Mann–Whitney rank sum tests and adjusted for multiple testing using Bonferroni correction.

### 2.8. Digital Droplet PCR Amplification of Resistance Genes

A PCR reaction was prepared according to BioRad’s 2× ddPCR Supermix for probes (no dUTP) instructions. Droplets were prepared and analyzed using a BioRad QX200 ddPCR system and evaluated with Quantasoft 1.7. The PCR reaction was conducted in a BioRad C1000 thermal cycler, following standard cycling settings. The primers used for the reactions can be found in Table 2. All analyses were performed in at least technical triplicates (*n* = 3–6).

## 3. Results

### 3.1. Composition of DNA Varies between Bacterial and Bacteriophage Compartments

**Figure 2:**
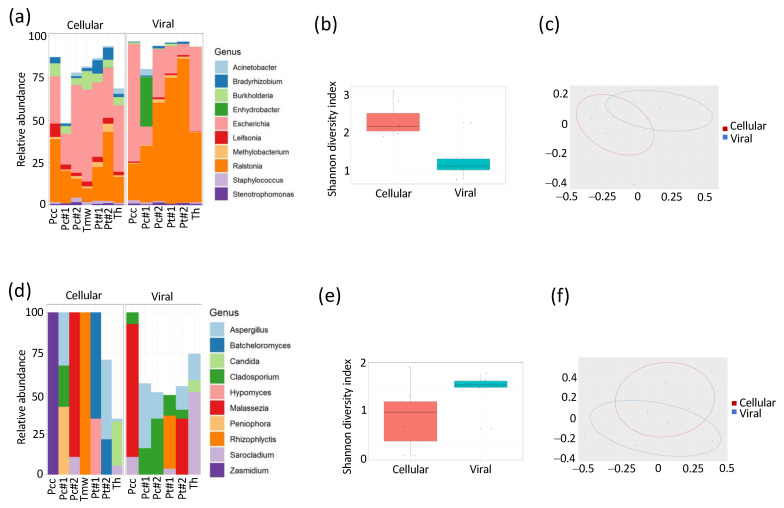
Microbial and fungal composition and diversity in water samples. Water samples were isolated from two general areas in Tanzania (P = Pongwe; T = Tanga City) from a recent cholera outbreak region (cc), city center (c), taps (t), hospitals (h) or medical ward taps (mw). The samples were divided into a bacterial and phage fraction, and the microbial composition determined using amplicon sequencing of conserved regions. Relative abundance of microbes is given in (**a**,**d**) for bacteria and fungi, respectively. Alpha (**b**,**e**) and beta (**c**,**f**) diversity of bacteria and fungi, respectively, are also given.

To mitigate the risk of infectious disease outbreaks and the spread of AMR through the environment, it is crucial to better understand local patterns and dynamics of microbial communities and AMR. To investigate the microbial composition in water samples from Tanzania during a cholera outbreak, we collected water from both taps and wells in different locations, both adjacent to and geographically far away from the site of the outbreak. Microbial components in the water were separated into cellular and viral fractions, after which DNA was extracted, and 16S and 18S amplicon sequencing was performed (Figure 2). Both the alpha (Shanon diversity index; 2b) and beta diversity (PcoA; 2c) differ significantly (*p* < 0.05) between the cellular (e.g., bacteria or fungi) and viral (e.g., bacteriophage) fractions. The relative abundance of bacteria and fungi within the cellular and viral fraction can be seen in Figure 2a,d, respectively. For bacteria, in particular, the genera *Bradyrhizobium*, *Burkholderia*, *Escherichia*, *Leifsonia,* and *Ralstonia* (ENA accession number: PRJEB58100; see Supplementary Table S1 for all accession numbers) were statistically different (*p* < 0.01). For fungi, only the genus *Cladosporium* was significantly different (*p* < 0.05) between the two compartments.

### 3.2. Water from Different Sources Is Heavily Contaminated with Classical Fecal, GI and Opportunistic Pathogens

**Figure 3:**
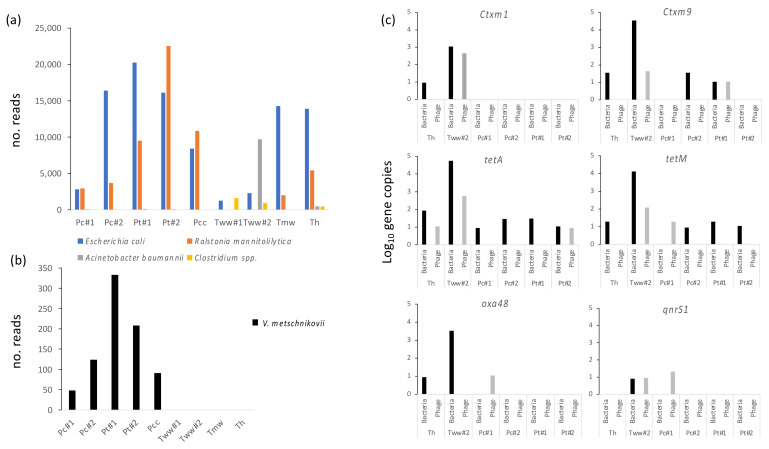
Water samples from different sources were collected and 16S amplicon sequencing performed on the purified bacterial DNA. Prevalence of several common GI-associated pathogens as well as opportunistic pathogens (**a**,**b**) are denoted based on number of reads in the amplicon data (total reads 37,291–140,684 depending on sample). (**c**) Log_10_ numbers of gene copies for different resistance genes from both bacterial and bacteriophage fractions.

All water samples contained significant levels of bacteria, most notably from human commensals and soil/environmental bacteria (Figure 2). However, all water sources, including hospital and town tap and well water, were heavily contaminated with fecal bacteria (e.g., *E. coli*) (Figure 3a), suggesting a high degree of fecal contamination in the drinking water. *E. coli* had the lowest general abundance in wastewater, likely due to the complexity of this material and its high diversity (e.g., lower reads for this specific species). Certain more common opportunistic pathogens, including *Acinetobacter baumannii*, were mainly identified in hospital outlets (Tww#2) but could also be identified in hospital tap water. Another common opportunistic pathogen is Ralstonia mannitolilytica, known to occasionally be the causative agent of sepsis [3]. No typical foodborne pathogens (*H. pylori*, *V. cholerae*) could be detected in any of the samples.

### 3.3. Unusual Vibrios Are Likely Causative Agents of a Cholera Outbreak

Due to the fact that the samples were collected during a cholera outbreak, specific emphasis was placed on investigating the presence of Vibrio in the samples. No V. cholerae could be detected in the samples isolated from the site of the cholera outbreak, nor in any of the other samples. However, the relatively less-studied Vibrio metschnikovii could be detected at high levels, specifically from the source of the cholera outbreak, as well as in all water sources in the vicinity of the outbreak (Figure 3a).

### 3.4. Several Antibiotic Resistance Genes Are Identified in high Numbers in Both Bacterial and Bacteriophage Fractions from Water Sources

To investigate the resistome profile of the water samples, three samples (Tww1, Pcc, Tmw) were analyzed through a nanopore metagenomics approach. All samples had several resistance genes towards tetracyclines and beta-lactams (Table 3). Furthermore, several resistance mechanisms towards macrolides, aminoglycosides, and phenicols could be identified. The high prevalence of tetracycline resistance genes was verified through ddPCR, with both tetA and tetM being identified in all investigated samples (Figure 3b). The highest abundance of resistance genes, as well as diversity, was identified in the hospital outlet wastewater (Tww#2), containing high levels of blaOXA-48.

## 4. Discussion

This study has successfully identified various microbial species in the water that could trigger infections and potentially lead to disease outbreaks. Microbial and fungal composition and diversity in water samples showed high variability. The findings reveal that the composition of DNA varies between bacterial and bacteriophage compartments. The differences strongly indicate that there is no significant carry-over of bacterial DNA to the phage compartment in the analysis. Instead, both compartments represent their own unique DNA reservoirs, making a comparison between cellular and viral fractions feasible. However, earlier evidence suggests that phages can also spread antimicrobial resistance (AMR) genes through generalized transduction.

The water samples displayed heavy contamination with fecal, gastrointestinal (GI), and opportunistic pathogens from various sources. The presence of fecal contamination in water sources is common in sub-Saharan countries, even within hospitals, and it has been estimated that several hundred million people in sub-Saharan countries lack access to safe and clean water supplies [31]. The prevalence of fecal contamination has been attributed to several factors related to water storage (time, protection), in-house water treatment, and how fecal matter is disposed of [32]. Such a high prevalence of fecal contamination has also been linked to both asymptomatic and symptomatic carriage of diarrhea-causing microbes [36], emphasizing the need for better drinking water practices. Specifically, it has been suggested that reducing fecal contamination on hands can contribute to improved water quality [37]. High levels of fecal contamination on hands have been associated with factors such as the educational level of parents in a household, access to advanced bathrooms, and the presence of infants in the household. Improved strategies for water, sanitation, and hygiene might help to reduce cholera outbreaks and eliminate local transmission.

The analysis reveals the unusual presence of *Vibrio metschnikovii*, a facultative aero-anaerobic gram-negative bacterium, in the water samples, which was likely the causative agent of a cholera outbreak. This bacterium is known as a rare human zoonotic pathogen that spreads through fecal–oral contamination, similar to *V. cholerae*. Unlike *V. cholerae*, however, very few clinical cases with this bacterium have been described, totaling less than 20 over the last 40 years [38]. Nevertheless, it has been shown to cause intestinal discomfort (e.g., diarrhea), skin infections, and occasionally blood infections [39]. It often exhibits a significant level of resistance to antibiotics [40]. *V. metschnikovii* has historically received limited attention, and only a few scientific reports have associated it with human pathology [38]. However, it has previously been isolated from cholera epidemics in Brazil, suggesting that it may be overlooked in several cases [41]. Most recently, it was also evaluated for its contribution to non-cholera vibriosis in Malaysia. However, out of the 270 reported cases, only two were assigned to *V. metschnikovii* [42]. Furthermore, the bacterium was not even isolated on a yearly basis during the 7-year surveillance program and was suggested to mainly be associated with foodborne diarrheal outbreaks. It would thus be important to follow up on these findings to further evaluate the significance of the findings and assess if *V. metschnikovii* is a common source of cholera-like outbreaks elsewhere in sub-Saharan countries. Furthermore, studies to better understand its virulence in humans and risk of infections would be needed to inform interventions to prevent and respond to future outbreaks linked to the pathogen.

The results demonstrate the presence of several antibiotic resistance genes in high numbers in both bacterial and bacteriophage fractions from water sources. The resistance genes *qnr*, *tetA/B*, *blaCTX-M*, and *blaOXA-48* have been reported as some of the more commonly identified resistance genes within Tanzania [43]. Outside wastewater, the most commonly identified resistance profiles were *tetA*, *tetM*, and *blaCTX-M9.* Similar to the prevalence of bacterial genes (e.g., 16S) within the viral fraction, the resistome significantly differed between the bacterial and viral fractions (Figure 3b). Specifically, *blaCTX-M1* is enriched in the bacteriophage fraction from wastewater, which aligns with previous findings [43]. In general, the bacterial community has a significantly higher abundance of resistance genes compared to the bacteriophage population (Figure 3b). However, the analysis does not allow for distinguishing between the presence of resistance genes on the actual phage vector (e.g., resistance genes integrated into the phage genome or in close proximity, allowing them to be packaged in the phage particle) and plasmidial or bacterial DNA carrying these resistance genes, being packaged in the phage capsid through the presence of specific sequence recognition sites (e.g., *cos* or *pac*). While plasmids are key drivers in the horizontal transfer of resistance genes, there are only a few reports of phages capable of integrating antibiotic resistance genes into their genome [44], suggesting that this is not a common mechanism for resistance spread. However, recent data has highlighted the existence of phage–plasmids (e.g., DNA structures that can exist as both self-replicating plasmids and integrative prophages) that are prone to carrying antibiotic resistance genes against a plethora of different classes (i.e., cephalosporins, carbapenems, aminoglycosides, fluoroquinolones, and colistin) [44]. It is thus imperative to further investigate this to better understand the molecular mechanisms underlying such enrichment of antibiotic resistance genes within bacteriophages.

## 5. Conclusions

In summary, our results demonstrate that bacteriophages play an important role in the transmission of resistance genes. Therefore, they should be considered a significant agent in the spread of antimicrobial resistance. Furthermore, our research highlights the importance of zoonotic pathogens in local outbreaks, suggesting a role for *V. metschnikovii* in fecal–oral transmission with diarrhea-inducing, cholera-like symptoms. Finally, our research underscores the need to continue working towards securing clean water, hygiene and sanitation for the global population, thereby reducing infectious diseases and the spread of antibiotic resistance in the context of the One Health concept. In many low and middle-income countries, water-based epidemiology needs improvement and integration as an essential component of the One Health implementation plan to enhance outbreak preparedness and response strategies, as well as measures to deter the spread of antimicrobial resistance.

## Figures and Tables

**Table 1:** Description of individually (*n* = 1) collected samples.

Sample	District	Village	GPS Location
Pcc	Pongwe	Manzara	5°08′38.5” S 38°58′29.2” E
Pt#1	Pongwe	Manzara	5°08′26.1” S 38°58′35.7” E
Pt#2	Pongwe	Manzara	5°08′26.1” S 38°58′35.7” E
Pc#1	Pongwe	Manzara	5°07′29.2” S 38°58′38.2” E
Pc#2	Pongwe	Manzara	5°07′29.2” S 38°58′38.2” E
Th	Tanga	Tanga	5°03′47” N 39°06′44” E
Tmw	Tanga	Tanga	5°03′47” N 39°06′44” E
Tww#1	Tanga	Tanga	5°03′47” N 39°06′44” E
Tww#2	Tanga	Tanga	5°03′47” N 39°06′44” E

**Table 2:** Primers used in the study, with primer pairs developed elsewhere.

Target	Forward Primer	Reverse Primer	Probe	Refs
16S	AGAGTTTGATCCTGGCTCAGGA	CGTGTTACTCACCCGTCCG	CGCTGGCGGCGTGCCTAATACATGC	[33]
*CTXM1*	ACAGTACAGCGATAACGTGG	GAATGGCGGTGTTTAACGTC	GCGGCCCGGCTAGCGTCACC	[34]
*CTXM9*	GACTGTGGGTGATAAGACCG	TGTTGCGGCTGGGTAAAATA	GCAGGGTCGTGCGCCGCTGG	[34]
*OXA48*	AAGTTACACGTATCGGAGCG	ACCAGCCAATCTTAGGTTCG	AGCCATGCTGACCGAAGCCAATGGTGA	[34]
*tetA*	TTGAACGGCCTCAATTTCCT	GATGAAGAAGACCGCCATCA	GCATGACCGTCGTCGCCGCCC	[33,34]
*tetM*	TGCAAGAAAAGTATCATGTGGAG	AAACCGAGCTCTCATACTGC	TGCCGCCAAATCCTTTCTGGGCTTCCA	[33,34]
*qnrS1*	GGGTGCATCACTGAAAGAGT	CCAGTGCTTCGAGAATCAGT	TGCCACGCCGAACTCGACGGTTTAGA	[34]
341F/806R	ACTCCTAYGGGRBGCASCAG	AGCGTGGACTACNNGGGTATCTAAT		[35]
G3 (V3-V4)	GCCAGCAGCCGCGGTAATTC	ACATTCTTGGCAAATGCTTTCGCAG		[35]
G4 (V3-V4)	CAGCCGCGGTAATTCCAGCTC	GGTGGTGCCCTTCCGTCAAT		[35]
G6 (V3-V5)	TGGAGGGCAAGTCTGGTGCC	ACGGTATCTGATCGTCTTCGATCCC		[35]

**Table 3:** Metagenomics of the resistome of individual water sources near Tanga, Tanzania.

Sample	Sample Source	Antibiotic Group	Resistance Gene
Tww1		Tetracycline	*tet(C, M, Q, W)*
		Beta-lactam	*blaTEM-1A, blaOXA, cfx(A3, A46)*
	Tanga, Wastewater	Macrolide	*erm(G), msr(D), med(A)*
		Aminoglycoside	*aac(6′), aph(2″), aph(3′)-Ia*
		Phenicol	*catQ*
Pcc	Pongwe, well	Tetracycline	*tet(C, M)*
		Beta-lactam	*blaTEM-1A, blaOXA-22*
Tmw		Tetracycline	*tet(C, K)*
		Beta-lactam	*blaTEM-1A, blaOXA-22*
	Tanga, tap		
	Medical ward	Aminoglycoside	*Aph(3′)-1a*
		Phenicol	*catA1*

## Data Availability

The data presented in the study are deposited in the European Nucleotide Archive (ENA) repository, accession number PRJEB58100.

## Supplementary Materials

The following supporting information can be downloaded at: https://www.mdpi.com/article/10.3390/microorganisms11102490/s1, Table S1: ENA sample accession numbers.
